# Predictive factors of self-medicated analgesic use in Spanish adults: a cross-sectional national study

**DOI:** 10.1186/2050-6511-15-36

**Published:** 2014-07-08

**Authors:** Pilar Carrasco-Garrido, Ana López de Andrés, Valentín Hernández Barrera, Isabel Jiménez-Trujillo, César Fernandez-de-las-Peñas, Domingo Palacios-Ceña, Soledad García-Gómez-Heras, Rodrigo Jiménez-García

**Affiliations:** 1 Department of Preventive Medicine and Public Health, Rey Juan Carlos University, Avda. Atenas s/n., Alcorcón, Madrid, Spain; 2 Department of Physical Therapy, Occupational Therapy, Rehabilitation and Physical Medicine, Rey Juan Carlos University, Alcorcón, Madrid, Spain; 3 Department of Nursing, Rey Juan Carlos University, Alcorcón, Madrid, Spain; 4 Department of Human Histology and Pathological Anatomy, Rey Juan Carlos University, Alcorcón, Madrid, Spain

## Abstract

**Background:**

Analgesics are among the most commonly consumed drugs by the world populations. Within the broader context of self-medication, pain relief occupies a prominent position. Our study was to ascertain the prevalence of self-medication with analgesics among the Spanish population and to identify predictors of self-medication, including psychological disorders, psychological dysfunction, mental health status, and sociodemographic and health-related variables.

**Methods:**

We used individualized secondary data retrieved from the 2009 European Health Interview Survey (EHIS) for Spain to conduct a nationwide, descriptive, cross-sectional pharmacoepidemiology study on self-medication with analgesics among adults (individuals aged at least 16 years) of both genders living in Spain. A total of 7,606 interviews were analysed. The dichotomous dependent variables chosen were the answers “yes” or “no” to the question In the last 2 weeks have you taken the medicines not prescribed for you by a doctor for joint pain, headache, or low back pain?” Independent variables were sociodemographic, comorbidity, and healthcare resources.

**Results:**

A total of 7,606 individuals reported pain in any of the locations (23.7%). In addition, analgesic consumption was self-prescribed in 23.7% (1,481) of these subjects. Forty percent (40.1%) of patients self-medicated for headache, 15.1% for low back pain, and 6.7% for joint pain. The variables significantly associated with a greater likelihood of self-medication of analgesics, independently of pain location were: age 16–39 years (2.36 < AOR < 3.68), higher educational level (1.80 < AOR <2.21), psychological disorders (1.56 < AOR < 1.98), and excellent/good perception of health status (1.74 < AOR < 2.68). In subjects suffering headache, self-prescription was associated with male gender (AOR 2.13) and absence of other comorbid condition (AOR 4.65).

**Conclusions:**

This pharmacoepidemiology study constitutes an adequate approach to analgesic self-medication use in the Spanish population, based on a representative nationwide sample. Self-prescribed analgesic consumption was higher in young people with higher educational level, higher income, smoker, and with psychological disorders and with a good perception of their health status independently of the location of pain.

## Background

Pain is a common human experience that is explained through a complex study of people within everyday environments and social context [1,2]. It is one of the main causes of morbidity worldwide affecting 10% to 30% of the European adult population; however, some studies have reported prevalence up to 50% [3-7]. In North America, the prevalence rate is 31% [8].

Pain is studied from different viewpoints. Associated psychological aspects can affect the consumption of analgesics. In fact, depression, anxiety and personality traits should be taken into consideration when analyzing how pain is perceived [9,10]. Self-care is practiced daily throughout by several people around the world. Self-medication is viewed as responsible involvement by private individuals in caring for themselves or members of their family [11]. This circumstance has been accentuated by the growing replacement of prescribed medicines by over-the-counter (OTC) remedies [12,13].

Pain relief occupies a prominent position within the broader context of self-medication [14,15]. Analgesics are the most commonly consumed drugs in the world, and it is estimated that millions of people in the United States, Australia, and Europe use OTC analgesics on a daily basis [16-18]. A Spanish study using data from the 2003 Spanish National Health Survey showed that, among individuals consuming analgesics, 39.4% were self-medicating [19].

Pain interrupts attention and interferes with daily life. Both psychological and social factors have been shown to predict outcome in individuals with chronic pain. In fact, depression is a common comorbidity, yet it is undiagnosed in clinical practice [20,21]. Given that psychiatric disorders are better diagnosed by structured interviews as part of the clinical practice, it is difficult to obtain reliable results in population-based studies. Some authors have used the generic SF-36 quality of life questionnaire in order to differentiate between patients with and without psychological disorders [22]. Further, health surveys are significantly commended as they collect valuable information related to musculoskeletal pain, which is not available from most other sources of information.

Therefore, the aims of the current study were: first, to ascertain the prevalence of self-medication with analgesics among the Spanish population; second, to identify variables associated to self-medication, including psychological disorders, mental health status, and socio-demographic and health-related variables.

## Methods

We conducted a nationwide, descriptive, cross-sectional epidemiologic study on self-medication among adults (individuals aged at least 16 years) of both genders living in Spain. This study was based on data obtained from the European Health Interview Survey for Spain (2009). As this study was conducted using de-identified public-use databases, it was not necessary to have the approval of an ethics committee.

### The 2009 European Health Interview Survey for Spain (EHISS)

The European Health Interview Survey (EHIS) was proposed by the European Commission to European Union Member States to create a health information system through comprehensive and coordinated surveys conducted in the European Statistical System under the responsibility of “Eurostat”. In such a way, all European Union States would share common guidelines for the survey modules (health determinants, health status, health care, background variables) and designs and based on a common questionnaire. The EHIS is implemented every five years, with the first wave completed between 2008 and 2009 [23].

In Spain, the EHIS was conducted by the National Statistics Institute (*Instituto Nacional de Estadística*, INE) under the aegis of the Spanish Ministry of Health and Social Affairs in 2009. This survey is a computer-aided home-based personal interview including nationwide representative sample of civilian, non-institutionalized population aged ≥ 16 years and residing in primary family dwellings (households) in Spain. Study subjects were selected by means of probabilistic multistage sampling, with the first-stage units being census sections and the second-stage units being primary family dwellings. Details of EHISS methodology are described elsewhere [24]. The data collection period ranged from April 2009 to March 2010. The analysis was performed in January 2013.

### Analgesic consumption definition

The dependent variable of the current study was created using the following two questions: “In the last two weeks have you taken those medicines prescribed for you by a doctor for joint pain, headache, or low back pain?”, or, “In the last two weeks have you taken the medicines not prescribed for you by a doctor for joint pain, headache, or low back pain?” An affirmative answer to the first question was considered as “prescribed analgesic use”. An affirmative answer to the second question was classified as “self-medicated analgesic use”. Total analgesic use included those with both prescribed and self-medicated drugs.

### Socio-demographic variables, lifestyle habits and co-morbid conditions

The following independent variables were included in the analysis. Main socio-demographic features including sex, age, marital status (married or not married), educational level (classified as no formal education, junior school or high school), occupational status (unemployed, employed or inactive), and monthly income.

Within lifestyle habits, smoking habit differentiated between current smokers or non-smokers. The alcohol consumption was measured using the question “Have you consumed alcoholic drinks in the previous 2 weeks?” Individuals were also asked for: “Do you practice any leisure time physical activity for at least 3 day per week? (Answer: active or inactive) Finally, the body mass index (BMI) was calculated from the self-reported body weight and height. Individuals with a BMI ≥ 30 were classified as obese.

Self-perceived health status was assessed with the following question: “How did you self-perceive your health status over the previous 12 months?” Subjects described health status as excellent, good, fair, poor or very poor. This variable was dichotomized into two: excellent/good or fair/poor/very poor self-perceived health status. Mental health was assessed with 2 indicators extracted from the Short-Form SF-36 quality of life questionnaire: psychological dysfunction and mental health status. Both indicators are obtained through 9 items which are scored from 0 to 100 points, where higher scores represent better mental health [25].

To identify individuals with associated chronic conditions, we used self-reported affirmative answer to the presence of any physician diagnosed concomitant diseases including hypertension, hypercholesterolemia, respiratory disease (asthma and chronic bronchitis), heart disease, diabetes, and cancer. We recorded whether patients had none, 1–2, or > 2 diseases. The presence of depression or anxiety (psychological disorders) was assessed using the following 2 questions: “Have you suffered from depression and/or anxiety the previous 12 months?”, and, “Has your medical doctor confirmed the diagnosis?”

### Statistical analysis

Self-medication patterns were identified using descriptive statistics of the main study variables by calculating the prevalence of total use and self-medication with analgesics for joint pain, headache, low back pain, and any pain localization during the years 2009–2010. Pearson χ^2^ test was used for the bivariate comparison of proportions.

Mean scores were also obtained for those variables from SF-36 (psychological dysfunction and mental health status) by calculating the difference in means between those who consume analgesics and those who did not for each of the pain localizations analyzed in the study, as well as the difference in means between those who consumed prescription analgesics and those who were self-medicated with analgesics for joint pain, headache, low back pain, and any localization.

To estimate the independent effect of the study variables on self-medication with analgesic drugs, we obtained the corresponding adjusted odds ratio (AOR) and the 95% Confidence Interval (95% CI) by means of multivariate logistic regression analysis. All variables showing a significant association in the bivariate analysis were included in the multivariate analysis, along with those variables that were considered relevant according to scientific literature. Four models were generated, as follows: one to identify factors associated with self-medication using analgesic drugs for joint pain; a second model to identify those factors predicting self-medication with analgesic drugs for headache; a third model for self-medication with analgesic drugs for low back pain; and a fourth logistic regression model to identify those factors associated with self-medication with analgesic drugs for pain at any pain localization. Estimates were made using the svy (survey commands) functions of the STATA program (STATA Corp, College Station, Texas, USA), which enabled us to incorporate the sampling design and weights into all statistical calculations (descriptive, χ^2^, logistic regression). Statistical significance was set at a 2-tailed *α* < 0.05.

## Results and discussion

Our results were based on data obtained from 7,606 subjects who answered the questions regarding consumption of analgesic medication (prescribed or self-medicated) in the 2 weeks immediately preceding the survey for any of the localizations analyzed. The sample represented 31.8% of subjects interviewed in the EHISS. Table 1 shows data on total analgesic consumption (with or without medical prescription) for joint pain headache, and low back pain and for any localization, according to socio-demographic characteristics, lifestyle, and health-related study variables. The results showed that 19.9% of the population consumed analgesics for low back pain, 12.6% for joint pain, and 10.9% for headache. The prevalence of consumption for pain at any localization was 39.8% for women and 23.5% for men (P < 0.05). Values for the consumption of analgesics were significantly higher when subjects were aged >65 years, had >2 chronic conditions, a chronic mental illness (anxiety and depression) or worse self-rated health status (P < 0.05).

**Table 1:** Prevalence of analgesic consumption (prescribed and self-medicated) for joint pain, headache, low back pain and any pain localization in Spanish adult according to socio-demographic variables, lifestyle, and health profile

		**Analgesic consumption for joint pain**		**Analgesic consumption for headache**		**Analgesic consumption for low back pain**		**Any localization**	
		**N**	**%**	**N**	**%**	**N**	**%**	**N**	**%**
Age*+Ɨǂ	16-39	176	2.3	738	10.6	1025	14.5	1662	23.4
	40-64	1232	12.8	1086	10.9	1963	20.7	3058	32.6
	>65	2009	33.5	590	9.6	1807	29.3	2886	47.4
Sex*+Ɨ	Male	820	7.2	688	7.2	1532	14.4	2445	23.5
	Female	2597	17.9	1726	14.5	3263	25.1	5161	39.8
Marital status*Ɨǂ	Not married	1641	10.8	1104	10.8	2224	18.8	3519	29.9
	Married	1776	14.0	1310	11.0	2571	20.7	4087	33.3
Educational level*+Ɨǂ	No formal education	1353	33.2	488	12.4	1306	32.1	1993	49.1
	Junior school	1158	17.7	549	9.6	1291	21.4	2093	34.8
	High school	906	6.2	1377	11.1	2198	16.6	3520	26.8
Occupational status*+Ɨǂ	Unemployed	209	8.44	269	11.58	446	18.80	671	27.97
	Employed	866	6.99	1205	10.91	1952	16.97	3155	27.59
	Inactive	2342	23.61	940	10.73	2397	25.15	3780	40.26
Monthly income*Ɨǂ	<850€	932	22.2	417	12.0	999	26.1	1540	40.3
	850-1400€	875	15.8	520	11.0	1180	22.6	1786	34.6
	>1400€	1094	9.6	1100	11.0	1935	18.5	3111	29.9
Smoking habit*+Ɨǂ	Non smoker	2935	14.9	1747	10.6	3683	20.8	5860	33.2
	Smoker	482	7.1	667	11.8	1112	17.7	1746	28.4
Alcohol consumption*+Ɨǂ	No	2552	12.9	1863	11.9	3572	20.6	5648	33.0
	Yes	610	9.6	434	8.2	931	16.1	1541	26.7
Physical activity*Ɨǂ	Inactive	2167	15.1	1340	10.9	2891	22.0	4484	34.3
	Moderate	1250	10.1	1074	10.9	1904	17.7	3122	29.3
Body Mass Index Kg/sq.m*Ɨǂ	<30	2148	10.3	1831	10.8	3392	18.4	5418	29.7
	≥30	850	22.1	407	12.1	952	26.4	1491	41.2
Self-assessment of health status*+Ɨǂ	Very good/Good	859	4.6	1302	8.8	2079	12.9	3632	22.7
	Fair/Poor/Very poor	2558	35.5	1112	17.1	2716	39.7	3974	57.7
Number of chronic conditions*+Ɨǂ	None	1839	9.0	1631	10.1	3035	17.3	4961	28.3
	1-2	1367	23.0	691	13.4	1550	27.1	2360	42.2
	>2	211	44.9	92	18.7	210	44.7	285	60.0
Psychological disorders*+Ɨǂ	No	2361	10.1	1784	9.6	3580	17.4	5911	28.8
	Yes	1056	31.8	630	21.3	1215	38.4	1695	54.1
Psychological dysfunction*+Ɨǂ (mean. se) [mean difference]		64.3 (0.5)	[-14.4]	68.2 (0.6)	[-9.88]	67.9 (0.4)	[-11.4]	69.8 (0.3)	[-10.48]
Positive mental health*+Ɨǂ (mean. se) [mean difference]		50.6 (0.5)	[-20.3]	57.5 (0.6)	[-2.18]	56.2 (0.4)	[-15.1]	58.6 (0.3)	[-14.18]
**TOTAL**		**3417**	**12.6**	**2414**	**10.9**	**4795**	**19.9**	**7606**	**31.8**

European Health Interview Survey (EHIS, 2009).

*Analgesic consumption for joint pain, +Analgesic consumption for headache.

ƗAnalgesic consumption for low back pain, ǂTotal analgesic consumption for any localization.

When pain location was analyzed, analgesic consumption was higher in women than men, individuals with >2 chronic conditions, subjects with psychological disorders, and those subjects reporting fair/poor/very poor health status (P < 0.05). It is noteworthy that consumption of analgesics was also higher in smokers and drinkers compared to non-smokers and no drinkers (P < 0.05). Table 1 summarizes the average scores for psychological dysfunction and mental health status. Subjects exhibiting joint pain who consume analgesics had the lowest scores in both domains of the SF-36.

Analgesic drug consumption was self-prescribed in 23.7% (1,481) of the sample. Table 2 shows the results of the 4 analyses performed to determine the proportion of analgesic self-medication for each of pain localizations according to socio-demographic characteristics, lifestyle and health-related variables. Men self-medicated their analgesic consumption than women (P < 0.05) and self-medication was significantly higher in the population aged 16–39 years than in remaining age groups (P < 0.05). Analgesic self-medication was more frequent in smokers and drinkers than in non-smokers and those subjects who not drinking. Additionally, more over-the-count medication was consumed by subjects reporting excellent/good health status and no chronic diseases (P < 0.05).

**Table 2:** Proportion of self-prescribed analgesic medication for joint pain, headache, low back pain and any localization in Spanish adult according to socio-demographic variables, lifestyle, health profile

		**Self-medication for joint pain**		**Self-medication for headache**		**Self-medication for low back pain**		**Self-medication for any localization**	
		**N**	**%**	**N**	**%**	**N**	**%**	**N**	**%**
Age*+Ɨǂ	16-39	42	19.8	406	57.2	270	28.0	655	41.5
	40-64	106	8.8	370	37.2	253	13.7	664	23.6
	>65	61	3.2	49	8.1	68	4.1	162	5.7
**Sex+Ɨǂ**	Male	58	7.5	349	55.2	225	17.0	587	27.8
	Female	151	6.4	476	32.8	366	14.1	894	21.3
**Marital status+Ɨǂ**	Not married	96	6.7	382	45.2	289	19.0	697	28.1
	Married	113	6.7	443	36.2	302	12.4	784	20.6
Educational level*+Ɨǂ	No formal education	38	2.9	47	11.3	52	4.6	115	6.4
	Junior school	59	5.4	135	29.5	84	7.6	255	14.4
	High school	112	12.7	643	50.7	455	23.2	1111	35.2
Occupational status*+Ɨǂ	Unemployed	16	8.15	98	36.62	58	14.10	151	23.20
	Employed	95	10.05	548	49.85	393	21.39	948	32.76
	Inactive	98	4.83	179	24.54	140	8.26	382	13.32
Monthly income*+Ɨǂ	<850€	28	3.1	69	21.2	53	6.4	139	11.4
	850-1400€	42	4.9	152	33.5	122	11.1	282	18.0
	>1400€	96	9.1	455	45.7	342	19.8	815	29.3
Smoking habit*+Ɨǂ	Non smoker	159	5.8	506	34.3	370	12.9	946	19.8
	Smoker	50	11.3	319	53.0	221	21.7	535	34.9
**Alcohol consumption+Ɨǂ**	No	146	6.2	605	39.0	428	15.1	1075	23.8
	Si	54	9.2	209	52.3	147	18.7	375	28.0
Physical activity*+Ɨǂ	Inactive	108	5.1	408	37.0	287	12.7	736	20.3
	Moderate	101	9.1	417	43.2	304	18.2	745	27.8
Body Mass Index Kg/sq.m*+Ɨǂ	<30	157	7.8	699	43.6	491	17.1	1238	27.0
	≥30	34	4.3	95	27.6	71	8.8	175	13.9
Self-assessment of health status*+Ɨǂ	Very good/Good	106	12.8	690	56.7	466	25.3	1173	36.2
	Fair/Poor/Very poor	103	4.5	135	15.9	125	5.7	308	9.7
Number of chronic conditions*+Ɨǂ	None	153	9.4	686	47.1	473	18.6	1206	28.7
	1-2	53	3.7	136	25.3	112	8.9	263	13.9
	>2	3	1.4	3	3.3	6	2.8	12	4.2
Psychological disorders t*+Ɨǂ	No	168	7.9	751	47.9	521	17.6	1326	27.0
	Si	41	3.8	74	13.6	70	6.8	155	10.6
**Psychological dysfunction+ǂ (mean. se) [mean difference]**		69.80 (2.1)	[5.8]	76.94 (0.8)	[14.6]	74.41 (1.0)	[7.7]	76.14 (0.6)	[8.3]
Positive mental health*+ǂ (mean. se) [mean difference]		56.76 (2.5)	[6.6]	67.55 (0.8)	[16.9]	65.54 (1.1)	[11.0]	66.56 (0.6)	[10.4]
**TOTAL**		**209**	**6.7**	**825**	**40.1**	**591**	**15.1**	**1481**	**23.7**

European Health Interview Survey (EHIS, 2009).

*Self-medication for joint pain, +Self-medication for headache.

ƗSelf-medication for low back pain, ǂSelf-medication for any localization.

When analgesic self-medication was analyzed by location, those experiencing headaches exhibited higher self-prescription (40.1%) than those suffering low back pain (15.1%) or joint pain (6.7%). The frequency of self-medication was higher in men than in women, in those without other chronic condition or without psychological disorders, and in those subjects reporting excellent/good health status (P < 0.05). The highest values for self-medication were always recorded for individuals suffering from headaches.

Table 2 also shows the average scores obtained for psychological dysfunction and mental health status for use of analgesics without prescription. Self-medication with analgesics for joint pain was the variable with the lowest scores in both domains of the SF-36.

The results of the multivariate logistic regression analysis (adjusted odd ratio) to identify predictors of self-medication with analgesics are summarized in Table 3. The variables that were significantly associated with a greater likelihood of self-medication of analgesics for joint pain were age (aged 16–39 years, OR 3.16 95% CI 1.75-5.71), high educational level (OR 2.21, 95% CI 1.31-3.71), psychological disorders (OR 1.56, 95% CI 1.01-2.46), and excellent/good perception of health status (OR 1.74, 95% CI 1.74-2.49). In subjects suffering headache, analgesic self-prescription was associated with age (aged 16–39 years, OR 3.68, 95% CI 2.25-6.03), male gender (OR 2.13, 95% CI 1.66-2.73) and higher educational level (OR 1.87, 95% CI 1.09-3.19), absence of other comorbid condition (OR 4.65, 95% CI 1.43-15.06), psychological disorders (OR 1.98, 95% CI 1.37-2.88), and excellent/good perception of health status (OR 2.38, 95% CI 1.74-3.26). Finally, the variables associated with a greater likelihood of self-medication for low back pain were age (aged 16–39 years OR 2.36, 95% CI 1.54-3.62), high educational level (OR 1.80, 95% CI 1.15-2.82), to be not married (OR 1.44, 95% CI 1.11-1.87), high monthly income (OR 1.70, 95% CI 1.14-2.54), smoking (OR 1.28, 95% CI 1.01-1.66), psychological disorders (OR 1.61, 95% CI 1.11-2.32) and excellent/good perception of health status (OR 2.68, 95% CI 1.97-3.65).

**Table 3:** Factors associated with analgesic self-medication among consumers with joint pain, headache, low back pain and any localization in Spanish adult

		**Self-medication for joint pain**	**Self-medication for headache**	**Self-medication for low back pain**	**Self-medication for any localization**
		**AOR, 95% CI**	**AOR, 95% CI**	**AOR, 95% CI**	**AOR, 95% CI**
**Age**	>65	1	1	1	1
	40-64	1.78 (1.18-2.70)	2.55 (1.63-3.99)	1.67 (1.12-2.47)	2.38 (1.82-3.09)
	16-39	3.16 (1.75-5.71)	3.68 (2.25-6.03)	2.36 (1.54-3.62)	3.71 (2.77-4.97)
**Sex**	Female	1	1	1	
	Male	NS	2.13 (1.66-2.73)	NS	NS
**Educational level**	No formal education	1	1	1	1
	Junior school	1.37 (0.81-2.33)	1.46 (0.85-2.53)	1.01 (0.62-1.62)	1.29 (0.93-1.80)
	High school/University	2.21 (1.31-3.71)	1.87 (1.09-3.19)	1.80 (1.15-2.82)	1.89 (1.37-2.62)
**Marital status**	Married	1	1	1	
	Not married	NS	NS	1.44 (1.11-1.87)	NS
**Monthly income**	<850€	1	1	1	1
	850-1400€	NS	NS	1.24 (0.82-1.89)	1.03 (0.77-1.38)
	>1400€	NS	NS	1.70 (1.14-2.54)	1.33 (1.01-1.75)
**Smoking habit**	Non smoker	1	1	1	1
	Smoker	NS	NS	1.28 (1.01-1.66)	1.34 (1.12-1.60)
**Self-assessment of health status**	Fair/Poor/Very poor	1	1	1	1
	Very good/Good	1.74 (1.22-2.49)	2.38 (1.74-3.26)	2.68 (1.97-3.65)	2.24 (1.81-2.78)
**Number of chronic conditions**	>2	1	1	1	1
	1-2	NS	3.45 (1.05-11.32)	NS	NS
	None	NS	4.65 (1.43-15.06)	NS	NS
**Psychological disorders**	No	1	1	1	1
	Yes	1.56 (1.01-2.46)	1.98 (1.37-2.88)	1.61 (1.11-2.32)	1.62 (1.24-2.11)

European Health Interview Survey (EHISS, 2009) for Spain.

Data are expressed as adjusted odds ratio (AOR) and 95% confidence intervals (95% CI).

NS: non-significant association.

When analyzed the variables associated with self-prescription of consumption of analgesics for the presence of pain in any localization, 6 variables were present: aged 16–39 years (OR 3.71, 95% CI 2.77-4.97), high educational level (OR 1.89, 95% CI 1.37-2.62), high monthly income (OR 1.33, 95% CI 1.01-1.75), smoking (OR 1.34, 95% CI 1.12-1.60), psychological disorders (OR 2.24, 95% CI 1.81-2.78), and excellent/good perception of health status (OR 1.62, 95% CI 1.24-2.11).

Analgesic drugs are worldwide used and are justified and necessary in cases of intense and frequent pain [14]. The results of our study showed that 23.7% of Spanish adults who consume analgesic medication use OTC drugs. Our data is higher than the 13.6% found in Finland [26], substantially lower than the 55% use of OTC analgesics found in Australian adults [15] but similar than in American adults (33%) [18]. It seems that self-prescription of analgesic drugs, i.e., OTC use is highly prevalent in populations across the world.

A common denominator associated with OTC analgesic drug consumption is the influence of gender. Although some studies using similar methodology than our study reported a higher probability of self-prescribed analgesic drug consumption in women [18,27,28], gender differences could be related to a higher prevalence of headaches in women since headaches are associated with greater consumption of both prescribed and OTC analgesics [29,30]. Nevertheless, Krymchantowski et al. found that men suffering chronic daily headache exhibited higher medication use than women [31]. It is possible that gender differences in self-prescribed analgesic consumption are related to several factors including specific co-morbid conditions and socio-cultural factors.

We found that subjects aged 19 to 39 years were almost 4 times as likely to use OTC analgesics for any pain location as elderly people. Current results also agree with previous studies. Stosic et al. showed a higher self-prescribed analgesic consumption and use of OTC drugs in Australian adults younger than 54 years old compared to those older than 55 years [15]. Wilcox et al. reported that American adults in the forties had a higher OTC analgesic consumption [18]. Current and previous results suggest that OTC drugs seem to be self-prescribed in a greater manner in the mid-age of the life, probably related to higher prevalence of pain syndromes and higher educational level. In fact, our results revealed that individuals taking OTC analgesics were young and highly educated (university level). Spanish university graduates were twice as likely to take OTC drug consumption as those with lower educational level. Klemenc-Ketis et al. found that 92% of Slovenian university students reported the use of self-medication [28]. These results agree with several published studies where OTC consumption was also associated with higher educational level [19,32,33]. Self-medication is more common among healthcare professionals (e.g. physicians, nurses, and pharmacists) than in the general population [34,35]. This can be related to the fact that healthcare professionals have university studies in almost all countries.

Further, a higher level of education can be related to a higher monthly income. When variable monthly income was analyzed, the results showed that subjects earning more than €1400 per month were more likely to take OTC analgesics, especially for back pain. These data are consistent with those reported in a recent study conducted in American adults where individuals with lower income were less likely to consume OTC non-steroidal anti-inflammatory drugs than those subjects with a higher income [33]. It is possible that a higher educational level involves higher monetary income and both variables are related to greater self-prescription consumption for pain.

Our results showed that smokers were more likely to self-medicate than the non-smoker population. This finding was similar to that previously reported by Hagen et al. in Norwegian adults who observed that smokers exhibited double risk of suffering from medication overuse headache [36]. Although experimental studies support that nicotine has analgesic properties, epidemiological evidence suggests that smoking is a risk factor for chronic pain [37].

Other factors associated with self-prescription of analgesic consumption were the absence of com-morbid conditions, only for those subjects suffering from headaches and excellent/good perception of health status. Previous studies had shown that people with chronic diseases or poor perception of health status are more likely to use analgesic medication [14,26,38] which is in accordance with our results. This observation may be related to cultural attitudes to pain [1] or the fact that individuals who self-medicate visit their doctor less often since their pain is less severe. This would be of particular interest in head pain, since headaches of lower/mild intensity can be usually controlled with analgesics. In fact, analgesics are usually consumed by subjects with sporadic pain symptoms. However, unnecessary analgesic use should be avoided since some drugs imply specific risks and, in the case or headaches, can produce medication overuse headache. The biggest challenge for health care professionals remains the provision of adequate pain management on an individual basis [24].

It is well accepted that pain is associated with psychological factors, particularly depression. Anxiety and depression have received the main attention, mostly in relation to recurrence and chronic pain in individuals with low back pain due to its relationship with physical disability and work absenteeism [9,39]. Our results showed a statistically significant association between the presence of psychological disorders (assessed with the SF-36 questionnaire) and self-medication for all the types of pain analyzed, although the strongest association was found in individuals suffering from headaches. Our results are in line with those previously found in Swedish people in who chronic headache was more prevalent in subjects who overused analgesics (39.8%) than in those who did not (18%) [40]. This study also observed that depression was significantly associated factor for analgesic overuse in general (OR 3.52, 1.46-8.52) [40].

In this context, community pharmacists are important members of health-care team. The pharmacists are probably the health professionals who are closest and most accessible to the patients and the general population. Their opinions related to health education have an important impact on the health of the population [41,42].

Although strengths of our study include large sample size, a randomly selected population, the employment of standardized survey, and training of the data collectors; there are also a number of possible limitations. First, since we used data from a cross-sectional survey, we cannot determine cause and effect relationships between the variables associated with self-prescribed medication use. Second, the conclusions drawn are difficult to generalize, because the survey has not been validated for drug use. Third, information obtained on interviews is subjective and may be subject to recall errors or a tendency of subjects to give socially desirable responses in the interviews. In addition, several of the data obtained from the interviews were self-reported which can induce a bias and, therefore, the prevalence of self-medication may be underestimated. Another possible limitation is that the survey rather than identifying specific active ingredients, it identifies groups of medications for specific diseases. Nonetheless, health surveys are significantly commended as they collect valuable information related to musculoskeletal pain problems, which is not available from most other sources of information.

## Conclusions

This pharmacoepidemiology study constitutes an adequate approach to analgesic self-medication use in the Spanish population, based on a representative nationwide sample. Our study found that 23.7% of Spanish adults use self-prescribed analgesic drug consumption to treat their pain with the highest incidence in headache (40.1%). We also observed that analgesic consumption without prescription seems to be higher in young people with higher educational level, higher monthly income, and smoker. In addition, subjects with psychological disorders and with a good perception of their health status also self-prescribed analgesic drugs. Further, self-prescribed analgesic consumption for headache was more frequent in men without co-morbid chronic diseases.

## Competing interests

### Financial competing interests

This study forms part of a project funded by the Fondo de Investigaciones Sanitarias (FIS) of the Carlos III Institute of Public Health.

### Non-financial competing interests

All of the authors declare no financial political, personal, religious, ideological, academic, intellectual, commercial or any other conflicts of interest, and none related to study interpretation.

## Authors’ contributions

(CGP): Researched data, wrote the manuscript, contributed to the discussion and reviewed/edited the manuscript. (LAA): Contributed to the discussion and reviewed/edited the manuscript. (HBV): Researched data and reviewed/edited the manuscript. (JTI), (FPC), (PCD) and (GGS): Contributed to the discussion and reviewed/edited the manuscript (JGR): Researched data, contributed to the discussion, and reviewed/edited the manuscript. All authors reviewed and gave their final approval of the version to be submitted

## Pre-publication history

The pre-publication history for this paper can be accessed here:

http://www.biomedcentral.com/2050-6511/15/36/prepub
